# Optogenetic/Chemogenetic Activation of GABAergic Neurons in the Ventral Tegmental Area Facilitates General Anesthesia via Projections to the Lateral Hypothalamus in Mice

**DOI:** 10.3389/fncir.2019.00073

**Published:** 2019-11-19

**Authors:** Lu Yin, Long Li, Jiao Deng, Dan Wang, YongXin Guo, XinXin Zhang, HuiMing Li, ShiYi Zhao, HaiXing Zhong, HaiLong Dong

**Affiliations:** Department of Anesthesiology and Perioperative Medicine, Xijing Hospital, The Fourth Military Medical University, Xi’an, China

**Keywords:** ventral tegmental area, general anesthesia, isoflurane, GABAergic neuron, lateral hypothalamus

## Abstract

The ventral tegmental area (VTA) reportedly regulates sleep and wakefulness through communication with the lateral hypothalamus (LH). It has also been suggested that adequate anesthesia produced by administration of chloral hydrate, ketamine, or halothane significantly reduces the GABAergic neuronal firing rate within the VTA. However, the exact effects on GABAergic neurons in the VTA and the mechanisms through which these neurons modulate anesthesia through associated neural circuits is still unclear. Here, we used optogenetic and chemogenetic methods to specifically activate or inhibit GABAergic neuronal perikarya in the VTA or their projections to the LH in Vgat-Cre mice. Electroencephalogram (EEG) spectral analyses and burst suppression ratio (BSR) calculations were conducted following administration of 0.8 or 1.0% isoflurane, respectively; and loss of righting reflex (LORR), recovery of righting reflex (RORR), and anesthesia sensitivity were assessed under 1.4% isoflurane anesthesia. The results showed that activation of GABAergic neurons in the VTA increased delta wave power from 40.0 to 46.4% (*P* = 0.006) and decreased gamma wave power from 15.2 to 11.5% (*P* = 0.017) during anesthesia maintenance. BSR was increased from 51.8 to 68.3% (*P* = 0.017). Induction time (LORR) was reduced from 333 to 290 s (*P* = 0.019), whereas arousal time (RORR) was prolonged from 498 to 661 s (*P* = 0.007). Conversely, inhibition of VTA GABAergic neurons led to opposite effects. In contrast, optical activation of VTA–LH GABAergic projection neurons increased power of slow delta waves from 44.2 to 48.8% (*P* = 0.014) and decreased that of gamma oscillations from 10.2 to 8.0%. BSR was increased from 39.9 to 60.2% (*P* = 0.0002). LORR was reduced from 330 to 232 s (*P* = 0.002), and RORR increased from 396 to 565 s (*P* = 0.007). Optical inhibition of the projection neurons caused opposite effects in terms of both the EEG spectrum and the BSR, except that inhibition of this projection did not accelerate arousal time. These results indicate that VTA GABAergic neurons could facilitate the anesthetic effects of isoflurane during induction and maintenance while postponing anesthetic recovery, at least partially, through modulation of their projections to the LH.

## Introduction

General anesthesia benefits tens of millions of patients who undergo surgery every year; however, the mechanisms through which general anesthesia is able to induce reversible unconsciousness is still one of the most baffling of puzzles. Elucidating these mechanisms is important for ensuring the safety of anesthesia and for developing more ideal and precise anesthetics. Deciphering the roles of key brain regions and neuronal types in anesthetic induction, maintenance, and arousal is an essential step toward these goals. It is known that key cellular targets of anesthetic agents are the GABAergic neurons in the brain (Hutt and Buhry, 2014). Although recent evidence has demonstrated that anesthetic agents may exert their effects through diverse neural circuitry in the brain (Franks, 2008), it is not thoroughly understood which specific brain regions are affected by and participate in GABAergic neuronal inhibition during general anesthesia.

The ventral tegmental area (VTA), especially its dopaminergic neurons, plays essential roles in many basic physiological and pathological functions, such as those associated with reward processing, negative emotional valence (Vanini et al., 2008; Tan et al., 2012), and sleep (Yu et al., 2019). During general anesthesia, activation of VTA dopaminergic neurons is sufficient to induce arousal from general anesthesia (Taylor et al., 2016). Lee et al. (2001) also found that the firing rate of VTA GABAergic neurons was significantly reduced under general anesthesia (Lee et al., 2001). In addition, a recent study indicated that GABAergic neurons in VTA regulate sleep and wakefulness through inhibitory projections to dentate gyrus (DG), lateral habenular nucleus (LHb), lateral preoptic area (LPO), and lateral hypothalamus (LH) (Yu et al., 2019).

With a great deal of axonal innervation from the VTA, the LH has been reported to regulate arousal, energy homeostasis, feeding behavior, and reward processing. Moreover, orexin (hypocretin) neurons in the LH play a vital role in the promotion and maintenance of wakefulness, both in general anesthesia and in sleep (Dong et al., 2006; Zhang et al., 2012; Cun et al., 2014; Zhou et al., 2018). Our previous studies have suggested that excitation of LH orexinergic neurons could facilitate emergence from propofol and isoflurane anesthesia via modulation of basal forebrain (BF) activity (Zhang et al., 2012, 2016).

Therefore, we hypothesized that VTA GABAergic neurons may modulate isoflurane anesthesia through their inhibitory projections to the LH. To test this hypothesis, we applied optogenetic and chemogenetic strategies, combined with electroencephalogram (EEG) analysis and behavioral tests, to investigate the role of VTA GABAergic neurons and their projections to the LH in mediating the induction, maintenance, and arousal stages of general anesthesia.

## Materials and Methods

### Animals

Vgat-Cre mice originated from Jackson ImmunoResearch Laboratories and were bred in our own lab. At the beginning of the viral microinjection, male mice aged 6–8 weeks were selected for behavioral testing, whereas 3-week-old mice were used for *in vitro* electrophysiological recording. The animals were housed in a specific-pathogen-free condition with constant temperature (24 ± 2°C) and humidity (60.0 ± 2.0%). Mice were kept on a 12-h:12-h light:dark cycle (lights on at 7:00 AM and lights off at 7:00 PM), with food and water supplied *ad libitum*. The experimental protocol used in this study was approved by the Ethics Committee for Animal Experimentation and conducted according to the Guidelines for Animal Experimentation in the Fourth Military Medical University.

### Anesthetic Administration

#### For Spectrum Analysis

For assessing the EEG spectrum change during optical stimulation of VTA GABAergic neurons and their axon terminals in the LH, we administered 0.8% (0.75 minimum alveolar concentration [Mac]) isoflurane to keep the mice at the sedation stage while avoiding burst suppression.

#### For Measurement of Burst Suppression Ratio

To determine the BSR from the EEG following optical manipulation of VTA GABAergic neurons and their axon terminals in the LH, we administered 1.0% (0.8 Mac) isoflurane to maintain the animals at an anesthetic plane, accompanied by steady burst suppression.

#### For Anesthesia Behavioral Testing

For assessing the effect of chemogenetic and optogenetic stimulation on behavior, including righting reflexes during induction and emergence from anesthesia, we administered 1.4% (1.0 Mac) isoflurane to maintain steady anesthesia.

Isoflurane was carried by pure O_2_ at a steady flow rate of 1.0 L/min.

### Virus Injection, Optic Fiber Implantation, and Electrode Fixation

Under 5.0% chloral hydrate (dissolved in normal saline, intraperitoneal) anesthesia, mice were fixed in the stereotaxic frame. The scalp was shaved and locally anesthetized with 2.0% lidocaine, followed by sagittal incision in the skin of the scalp.

Viral injection for optogenetic experiments included unilateral rAAV-Ef1α-DIO-ChR2-mCherry or bilateral rAAV-Ef1α-DIO-eNpHR-mCherry (BrainVTA, China), which were injected into the VTA (anterior–posterior [AP] −3.5 mm, medio-lateral [ML] ± 0.4 mm, and dorso-ventral [DV] −4.0 mm) at a rate of 50 nl/min (200 nl/side) for inhibition and excitation, respectively. After injection, the tip of the micropipette was left in place for 10 min and then retrieved slowly. The optical fiber and electrode implantations were conducted afterward.

For chemogenetic experiments, AAV-Ef1α-DIO-hM3Dq-mCherry, AAV-Ef1α DIO-hM4Di-mCherry, or rAAV-Ef1α-DIO-mCherry (BrainVTA, China) were bilaterally injected into the VTA as described above. After microinjection, the incision was sutured.

Optical fibers (2.5/1.25 mm in diameter, 200-μm fiber optic cable, numerical aperture [NA] = 0.37) were implanted into the VTA (AP −3.5 mm, ML ± 0.4 mm, DV −3.95 mm) or the LH (AP −1.75 mm, ML ± 0.9 mm, and DV −5.05 mm) for stimulation of VTA GABAergic neuronal cell bodies or axon terminals.

Three stainless steel screws were also implanted in the skull at three sites to allow for attachment of EEG electrodes as previously described (Li et al., 2019). Finally, all the screws and fiber were secured to the skull with dental cement. After surgery, the EEG recording and behavioral testing were conducted at least 3 weeks later for animal recovery and viral infection.

### Optical Stimulation and Electroencephalogram Recording

Mice were first habituated to a plexiglass cylinder (45 cm in length, 30 cm in diameter) with the laser stimulator and EEG recording cable connected to their skulls. EEGs were recorded with a sampling frequency of 1,000 Hz and a bandpass set at 0.3–50 Hz using a PowerLab 16/35 amplifier system (PL3516, ADInstruments) and LabChart Pro V8.1.13 (MLU60/8, ADInstruments).

**FIGURE 1:**
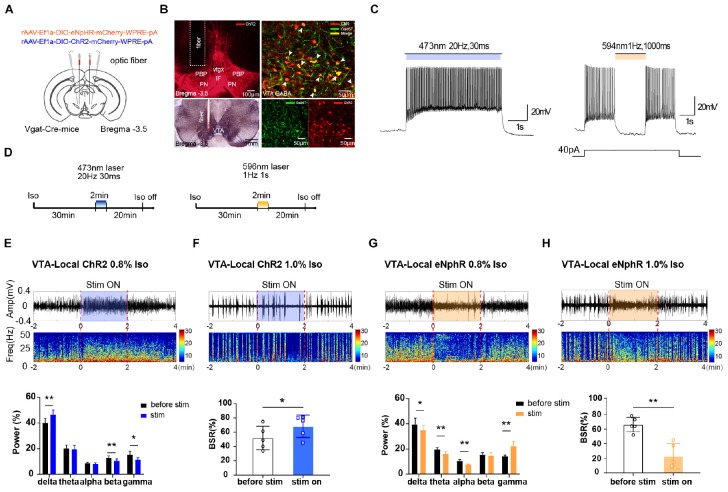
The effect of optogenetic modulating VTA GABAergic neurons during isoflurane anesthesia on EEG. **(A)** Schematic diagram of optogenetic virus injection and optical stimulation sites. **(B)** Viral expression (mCherry, red) in GABAergic neurons in the VTA and co-labeling with GABA (GAD67 immunofluorescence, green). White arrows indicate the Gad 67 labeling in the cytoplasm of GABAergic neurons. White lines in the left picture outline the trace of an implanted fiber. **(C)** Diagram of electrophysiology recording for virus function examination in Vgat-Cre mice. ChR2 virus was irradiated by (473 nm) for 420 s at frequencies of 20 Hz; eNpHR virus by laser irradiation (594 nm) at a frequency of 1 Hz for 1000-ms duration. **(D)** Diagram showing the protocol of optical activation and inhibition of VTA GABAergic neurons during the inhalation of isoflurane. The colored line represents the time span of laser on (blue for 473-nm laser and yellow for 596-nm laser). Average spectrum (top row) and spectral percentage (bottom row) changes during optical activation **(E)** and inhibition. **(G)** The time span from 2 before 2 min after the end of light activation. Average spectrum and BSR changes during optical activation **(F)** or inhibition **(H)**. The time span is the same as in **(D)** and **(F)**. ^∗^*P* < 0.05, ^∗∗^*P* < 0.01. VTA, ventral tegmental area; EEG, electroencephalogram.

For spectral analysis, 0.8% isoflurane was delivered constantly for 30 min. When the EEG signal stabilized, 2 min of optical manipulation was applied. A train of blue laser pulses (473 nm, 20 Hz, 30 ms, and 15 mW from the fiber tip) was delivered to induce activation, whereas a train of yellow laser pulses (594 nm, 1 Hz, 1 s, and 10 mW from the fiber tips) was delivered to induce inhibition (Figure 1D). Stimulation parameters were the same for both VTA GABAergic cell bodies and the axon terminals in the LH. During the experiment, isoflurane concentration was monitored using Philips G60, whereas EEG signal was constantly recorded for 30 min after optical stimulation.

For BSR calculations, EEG signal collection and optical stimulation parameters were the same as was described above.

For behavioral testing, the mice were constantly optically stimulated (activation, 473 nm, 20 Hz, and 30-ms duration; or inhibition, 594 nm, 1 Hz, and 1-s duration) every 60 s with 30-s interval. During induction, the opto-stimulation was administered at the start of isoflurane delivery until mice achieved LORR. During emergence from anesthesia, mice were optically stimulated from the time isoflurane administration ceased until the mice achieved RORR.

### Electroencephalogram Analysis

The signals were post-processed using custom MATLAB (MathWorks, Natick, MA, United States) scripts. The parameters were set as follows: non-equispaced fast Fourier transform (nFFT) = 2,048; sampling frequency (Fs) = 1,000; window function (windows) = Hanning; window overlaps the number of points (overlap) = (length [windows])/2. In the spectrum, the power was mainly distributed in the frequency band with dark and warm colors. The frequency bands were composed of delta (δ: 0.3–4 Hz), theta (θ: 4–10 Hz), alpha (α: 10–15 Hz), beta (β: 15–25 Hz), and gamma (γ: 25–50 Hz) waves. The average spectral power percentage over an interval spanning the 2 min before and during stimulation was recorded for spectral statistical analysis.

For BSR calculations, EEG data were scored and divided into burst and suppression portions by basic voltage lines (Li et al., 2019). The voltage interval was set according to the amplitude of the suppression waves measured from the mice. If the amplitude of the EEG was less than the interval threshold, the amplitude was categorized as a suppression event and assigned a value of 1, whereas signals whose amplitudes were greater than the interval threshold were categorized as burst events and assigned a value of 0. The minimum duration of the suppression wave was 0.5 s. Finally, the BSR was calculated by dividing the frequency of events assigned a value of 0 by the frequency of events assigned a value of 1 for 2 min before and during the optical stimulation.

### Estimation of Induction and Emergence Times

For chemogenetic experiments, clozapine N-oxide (CNO, 3 mg/kg in saline) or saline vehicle (the same volume) was injected intraperitoneally before the animal was anesthetized. After 30 min of free exploration, animals were anesthetized (1.4% isoflurane) in the cylinder. The cylinder was gently rotated by 90° every 10 s until the mouse experienced a LORR and all its limbs were oriented in an upward direction. The duration from onset of isoflurane exposure to LORR was recorded as induction time.

After 30 min of exposure, the isoflurane was shut off and the cylinder was rotated as described above to assess the duration of time before the righting reflex (RORR) reappeared, defined as the moment when mice could independently turn from the supine position with at least three paws reaching the bottom of the cylinder.

We then tested the effect of chemogenetic modulation of VTA GABAergic neurons on the EC_50_ of isoflurane anesthesia. During induction, at the very beginning, the isoflurane vaporizer was turned on to 0.2%, and then the concentration was increased by 0.2% every 15 min until all the animals reached LORR. At every 0.05% increment of detector reading, animals that had achieved LORR were documented. During emergence, mice had been anesthetized with 1.4% isoflurane for 30 min, and then the isoflurane concentration was reduced by 0.2% every 15 min. The number of animals that had reached RORR was recorded at every 0.05% reduction in detector reading. In either experiment assessing LORR or RORR, CNO was administered 30 min before isoflurane delivery.

For optogenetic experiments, mice were laser stimulated until LORR or RORR was achieved, as previously described.

### Slice Recording and Optical Parameter Verification

After 3 weeks of viral expression, mice brains were collected and immediately submerged in an ice-cold solution containing (in mM) 124 NaCl, 25 NaHCO_3_, 2.5 KCl, 1 NaH_2_PO_4_, 2 CaCl_2_, 2 MgSO_4_, and 37 glucose, saturated with 95.0% O_2_/5.0% CO_2_. Brains were mounted in a vibratome (Leica VT1200S) and kept submerged, ice cold, and oxygenated. Coronal slices containing the VTA were cut at 300 μM and incubated for 45 min at 35°C in artificial cerebrospinal fluid (ACSF) containing (in mM) 124 NaCl, 24 NaHCO_3_, 3.8 KCl, 1.2 NaH_2_PO_4_, 1 MgCl_2_, 2.5 CaCl_2_, and 10 glucose, saturated with 95.0% O_2_/5.0% CO_2_. Then slices were maintained at room temperature with continuous oxygenation prior to experimentation. Individual brain slices were transferred to the recording chamber and perfused continuously with oxygenated ACSF (1.5–2 ml/min) at room temperature.

Whole-cell recording was performed using micropipettes prepared from borosilicate glass capillaries (1.5 mm outer diameter [OD], 1.1 mm inner diameter [ID]) using a horizontal puller (P-97, Sutter Instruments), with resistances between 4 and 6 MΩ. The pipette solution contained (in mM) 130 K-gluconate, 4 KCl, 1 MgCl_2_, 10 hydroxyethyl piperazineethanesulfonic acid (HEPES), 0.3 egtazic acid (EGTA), 4 Mg-ATP (adenosine triphosphate), and 0.3 Na-GTP (guanosine-5-triphosphate) (pH = 7.4). Current clamp was used to assess the electrophysiological characteristics in response to activation or inhibition of GABAergic neurons in the VTA.

### Immunofluorescent Labeling to Confirm Viral Transfection and Optical Fiber Implantation Success

After being anesthetized with pentobarbital (100 mg/kg, i.p.), mice were transcardially perfused with 20 ml of cold saline, followed by 20 ml of 4.0% paraformaldehyde in phosphate-buffered saline (PBS, pH = 7.4). Mice brains were dissected, postfixed in paraformaldehyde for 2 h, and cryoprotected (in 30% sucrose in PBS) for 72 h at 4°C. Later, the midbrains (containing the VTA) were frozen and coronally sliced at 40 μm with a cryostat (Leica, CM1900 freezing microtome, Germany). Slices were rinsed with PBS and blocked with 5.0% normal donkey serum (NDS) with 0.3% Triton X-100 in PBS (PBST). Then, a glutamate decarboxylase (Gad67) antibody (mouse 1:400, MAB5406, Millipore, United States) was applied in 2.5% NDS in PBST overnight at 4°C. Sections were washed again thoroughly and incubated in a secondary antibody (Jackson ImmunoResearch Laboratories 715-545-150 1:500, donkey-anti-mouse IgG, biotin-conjugated) for another night at 4°C. After being washed with PBS, slices were incubated in streptavidin AF 488 (1:500 diluted in 2.5% NDS with PBST) for 2 h, and then sections were further rinsed, mounted, and cover slipped. Images were captured using a laser confocal fluorescent microscope (FV1200, Olympus, Japan). If the viral transfection was poor or the optical fiber site was incorrect, associated data for that animal were discarded.

### Statistical Analysis

Prism8 (GraphPad) was used for statistical analysis. Parametric data are presented as mean ± SD, unless otherwise stated in the figure legends. Unpaired *t*-test or two-way ANOVA followed by Tukey’s *post hoc* test was performed to assess the statistical differences. All tests were two-tailed. *P-*values are shown when they are less than 0.05.

## Results

### Optogenetic Modulation of Ventral Tegmental Area GABAergic Neurons Affected Electroencephalogram Readings During General Anesthesia

To verify the role of VTA GABAergic neurons in mediating anesthesia, excitatory or inhibitory optogenetic viruses were injected into the VTA of Vgat-Cre mice with optical fibers implanted (Figure 1A). The immunolabeling results verified that the VTA neurons transfected with optogenetics viruses (ChR2-mCherry, red) were GABAergic neurons (anti-GAD67, green), and that the optical fibers were implanted at the correct location (Figure 1B). VTA GABAergic neurons were successfully activated or inhibited by optical stimulation in the brain slices. In Vgat-Cre mice, neurons expressing ChR2 were activated by laser irradiation (473 nm) for 5 s at frequencies of 1, 10, 20, 30, and 50 Hz, with a 30-ms pulse width. On the basis of our pilot results and references, we chose 20 Hz for 30 ms as the ideal parameter for optic stimulation because high frequencies induced firing loss during light stimulation. Neurons expressing the eNpHR virus were stimulated by laser (594 nm) at a frequency of 1 Hz for 1-s duration (Figure 1C).

Optical activation of VTA GABAergic neurons significantly increased delta power percentages from 40.0 ± 3.1% to 46.4 ± 3.4% (*n* = 5, *P* = 0.006, *t* = 5.32) while reducing those of beta and gamma waves (Figure 1E). On the contrary, optical inhibition of VTA GABAergic neurons significantly elevated the gamma power percentages from 14.45 ± 1.3% to 21.9 ± 3.4% (*n* = 5, *P* = 0.007, *t* = 5.17) while decreasing the delta, theta, and alpha power percentages (Figure 1G). These results confirmed that activation of VTA GABAergic neurons could further inhibit brain activity during anesthesia.

For BSR calculation, under 1.0% isoflurane anesthesia, activation of VTA GABAergic neurons led to a significant BSR increase from 51.8 ± 14.7% to 68.3 ± 14.0% (*n* = 5, *P* = 0.017, *t* = 3.95) (Figure 1F), whereas inhibition resulted in BSR reduction from 66.1 ± 8.9% to 22.7 ± 15.4% (*n* = 5, *P* = 0.001, *t* = 7.99) (Figure 1H). Although burst suppression is always associated with delirium and postoperative cognitive dysfunction (POD), stable BSRs could partly reflect the depth of anesthesia during maintenance. Radtke et al. (2013) reported that the mean intraoperative BSR is around 7.1–8.8%. If BSR is over 40.0% (Radtke et al., 2013), it will be directly and linearly related to the bispectral index (BIS = 50 − BSR/2) (Bruhn et al., 2000). Moreover, BSR is widely used in animal experiments to evaluate whether the approaches can reverse stable, deep anesthesia, when a larger proportion of the cortex is inactive (Brown et al., 2010). The BSR alteration, in addition to the observed spectral changes, indicated that VTA GABAergic neurons mediate isoflurane anesthesia maintenance.

### Chemogenetic Modulation of Ventral Tegmental Area GABAergic Neurons Affects the Induction and Emergence Time From Isoflurane Anesthesia

**FIGURE 2:**
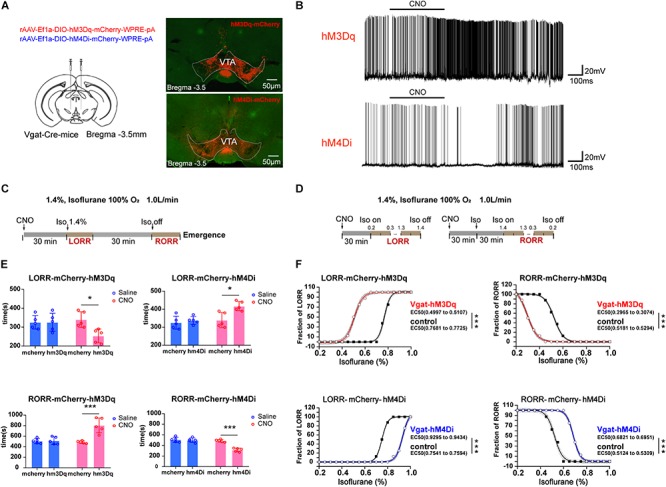
Influences of chemogenetic modulation of VTA GABAergic neurons on anesthetic induction and emergence. **(A)** Schematic diagram of the chemogenetic virus injection site (left) and viral expression (hM3Dq and hM4Di) in GABAergic neurons in the VTA (right). **(B)** Diagram of electrophysiology recording for virus function examination of hM3Dq and hM4Di by CNO administration. **(C)** Schematic diagram of the experimental protocol to observe the effects of VTA GABAergic neuronal activities on time of LORR or RORR under 1.4% isoflurane anesthesia. **(D)** Schematic diagram of the experiment for calculating EC_50_ of isoflurane on LORR or RORR. **(E)** Statistical results for the effects of modulating VTA GABAergic neuron activities on time of LORR and RORR under 1.4% isoflurane anesthesia. **(F)** Statistical results for the effects of modulating VTA GABAergic neuron activities on EC_50_ of LORR and RORR under the condition where isoflurane was given progressively. ^∗^*P* < 0.05, ^∗∗∗^*P* < 0.001. VTA, ventral tegmental area; EEG, electroencephalogram; CNO, clozapine N-oxide; LORR, loss of righting reflex; RORR, recovery of righting reflex.

To further investigate the effect of VTA GABAergic neurons on anesthesia, behavioral testing was performed. In these experiments, chemogenetic viruses were microinjected into the VTA of Vgat-Cre mice, and the injection sites were confirmed by assessing the distribution of mCherry fluorescence under a confocal microscope (Figure 2A).

With the use of CNO to activate VTA GABAergic neurons, the duration to LORR was significantly shortened from 340 ± 41.83 to 254 ± 36.47 s [*F*(1, 16) = 5.569, *P* = 0.031], whereas the duration to RORR was prolonged from 488 ± 23.87 to 806 ± 139.92 s [*F*(1, 16) = 17.58, *P* < 0.0007]. Moreover, inactivation of VTA GABAergic neurons could reverse their anesthesia-promoting effect by slowing down induction from 340 ± 41.83 to 418 ± 25.88 s [*F*(1, 16) = 5.223, *P* = 0.036] and accelerating time to emergence from anesthesia from 488 ± 23.87 to 316 ± 34.35 s [*F*(1, 16) = 22.91, *P* = 0.0002]. The injection of CNO significantly increased the firing rate of VTA GABAergic neurons, and this effect could be washed out (Figure 2B). Schematic protocol of anesthesia behavior test is showed in Figure 2C. CNO does not affect LORR or RORR in control groups (Figure 2E). All these results confirmed that VTA GABAergic neurons could facilitate general anesthesia.

To identify a role of VTA GABAergic neurons on isoflurane anesthesia sensitivity, progressive delivery or withdrawal of isoflurane was applied. Schematic protocol of anesthesia sensitivity experiment is showed in Figure 2D. We found that the EC_50_ of isoflurane in terms of LORR was reduced from 0.78 to 0.51% in the activation group (hM3Dq) and augmented from 0.76 to 0.94% in the inhibition group (hM4Di) (Figure 2F). On the other hand, VTA GABAergic neuron activation resulted in a significant EC_50_ reduction in terms of RORR from 0.52 to 0.30% (*n* = 8, *P* < 0.0001), whereas the inhibition increased EC_50_ of isoflurane on RORR from 0.52 to 0.69% (*n* = 8, *P* < 0.0001) (Figure 2F), indicating that VTA GABAergic neurons could enhance isoflurane anesthesia sensitivity.

### Ventral Tegmental Area GABAergic Neurons Promote Anesthesia Through the Lateral Hypothalamus

**FIGURE 3:**
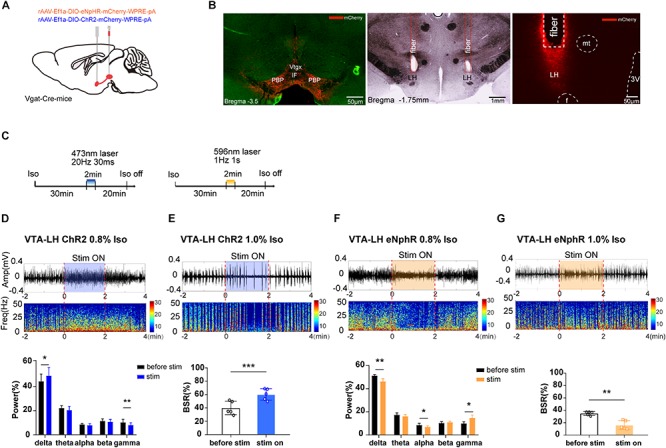
The effect of optogenetic modulation of VTA–LH GABAergic projection on EEG during anesthesia. **(A)** Schematic diagram of optogenetic virus injection and stimulation sites. **(B)** Fluorescent confirmation of virus expression (mCherry) in GABAergic neurons in the VTA and LH (Vgat-Cre mice) and the trace of implanted fibers in the LH. **(C)** Diagrams showing the protocols for optical activation and inhibition of VTA GABAergic terminals in the LH during the isoflurane anesthesia. The colored line represents the time span of laser on (blue for 473-nm laser and yellow for 596-nm laser). Average spectrum (top row) and spectral percentage (bottom row) changes during optical activation **(D)** and inhibition. **(F)** The time span from 2 min before to 2 min after the end of light activation. Average spectrum and BSR changes during optical activation **(E)** or inhibition **(G)** The time span is the same as in **(D)** and **(F)**. ^∗^*P* < 0.05, ^∗∗^*P* < 0.01, and ^∗∗∗^*P* < 0.001. VTA, ventral tegmental area; LH, lateral hypothalamus; EEG, EEG, electroencephalogram; CNO, clozapine N-oxide; LORR, loss of righting reflex; RORR, recovery of righting reflex; BSR, burst suppression ratio.

Though VTA GABAergic neurons could innervate multiple brain areas to regulate sleep and wakefulness, the LH receives extensive projections from the VTA and is involved in sleep and anesthesia regulation (Yu et al., 2019). Therefore, we assessed the role of VTA–LH GABAergic projections in mediating general anesthesia. As shown in Figure 3A, optogenetic viruses were administered into the VTA, and optical fibers were implanted into the LH for manipulation of the VTA GABAergic terminals. Associated viral expression and the positions of the optical cannula were also verified as described above (Figure 3B). The schematic protocol of optogenetic stimulation is showed in Figure 3C.

As we expected, a 2-min activation of VTA–LH GABAergic terminals during the 0.8% isoflurane maintenance period could significantly increase delta power percentage from 44.18 ± 5.41% to 48.83 ± 6.03% (*n* = 5, *P* = 0.014, *t* = 4.16) while reducing gamma power from 10.20 ± 2.32% to 8.04 ± 1.64% (*n* = 5, *P* = 0.006, *t* = 5.41). However, the beta power percentage did not change (Figure 3D). Optogenetic inhibition of VTA GABAergic terminals in the LH reduced delta and alpha power percentages and increased the gamma power percentage from 9.95 ± 1.52% to 14.65 ± 2.64% (*n* = 5, *P* = 0.018, *t* = 3.85), with no effect on theta power (Figure 3F). Furthermore, activation of VTA–LH GABAergic projections evidently increased BSR from 39.9 ± 8.97% to 60.20 ± 7.70% (*n* = 5, *P* = 0.0002, *t* = 13.03) under 1.0% isoflurane anesthesia (Figure 3E), whereas inhibition of the projections significantly reduced BSR from 34.90 ± 2.73% to 16.31 ± 6.63% (*n* = 5, *P* = 0.002, *t* = 7.31) (Figure 3G). All these results suggest that the VTA–LH GABAergic projections may be the principle pathway through which the anesthesia-promoting effect of VTA GABAergic neurons is mediated.

**FIGURE 4:**
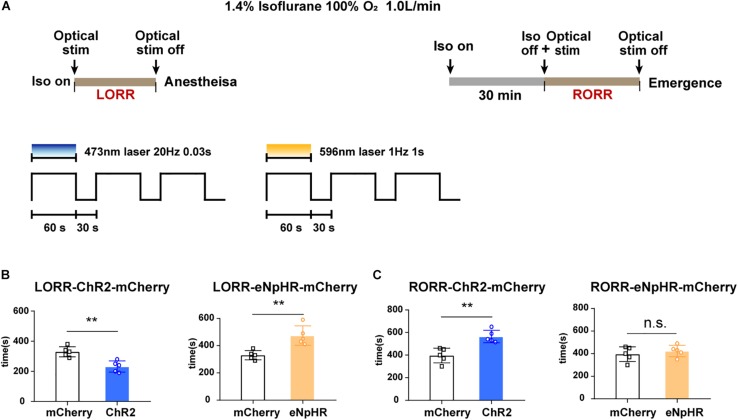
Optogenetic stimulation of VTA–LH GABAergic projection regulates anesthetic induction and recovery. **(A)** Diagrams showing the protocol of optical activation and inhibition of VTA GABAergic neurons in terminals in the LH on the induction of or emergence from isoflurane anesthesia. **(B)** Effects of activating or inhibiting VTA GABA-LH projections on the time of LORR. **(C)** Effects of activating or inhibiting VTA GABA-LH projections on the time of RORR. VTA, ventral tegmental area; LH, lateral hypothalamus; LORR, loss of righting reflex; RORR, recovery of righting reflex. ^∗∗^*P* < 0.01.

To further test the conclusion, the durations of LORR and RORR were recorded during continuous 1.4% isoflurane anesthesia in different groups of animals. The experimental design is shown in Figure 4A. We discovered that constantly optogenetic activation of VTA–LH GABAergic terminal significantly reduced the LORR time from 330 ± 33.1 to 232 ± 33.1 s (*P* = 0.002, *t* = 4.37, df = 8), whereas inhibition of VTA–LH GABAergic projections prolonged LORR from 330 ± 33.1 to 474 ± 63.75 s (*P* = 0.004, *t* = 4.08, df = 8) (Figure 4B). For anesthesia emergence, activation of VTA–LH GABAergic terminals significantly increased RORR from 396 ± 65.04 to 565 ± 55.22 s (*P* = 0.002, *t* = 4.43, df = 8). However, we did not detect a statistical difference in RORR after anesthesia following the inhibition of the projection (Figure 4C).

## Discussion

In this study, we verified that VTA GABAergic neurons and their projections to the LH region are involved throughout the induction, maintenance, and emergence from isoflurane anesthesia. Both chemogenetic and optogenetic activation of VTA GABAergic neurons and their LH projections facilitated isoflurane induction, increased depth of anesthesia during maintenance, and prolonged emergence time from anesthesia, whereas inhibition of these targets had contrasting these effects on anesthesia.

A role of GABAergic neurons in anesthesia has long been proposed (Vanini et al., 2008; Ferron et al., 2009; Zecharia et al., 2012; Zurek et al., 2012; Kuki et al., 2015); studies have shown that isoflurane decreases the amplitude of GABA_A_-R-mediated inhibitory postsynaptic current (IPSC) and prolongs the decay of the GABA-evoked response. At the same time, isoflurane could also slow the rate of GABA dissociation from GABA_A_-Rs, leading to an increase of the GABA-induced charge transfer and enhancement of GABAergic inhibition (Hapfelmeier et al., 2001). It was believed that isoflurane inhibited GABAergic neurons in the cerebral cortex, hippocampus, striatum, and spinal cord (Westphalen et al., 2011). However, Jiang-Xie et al. (2019) recently confirmed that anesthetics diminished the c-Fos expression throughout the brain, except a cluster of anesthesia-activated neurons (AANs) in the ventral region of the hypothalamus. GABAergic neurons account for 2.3 and 45.2% of all AANs in the SON and paraSON (including the ventral preoptic nucleus [VLPO] and POA), respectively (Moore et al., 2012; Jiang-Xie et al., 2019).

In this study, we investigated the role of GABAergic neurons of the VTA in mediating general anesthesia. Although dopaminergic neurons are the most abundant neuronal subtype in the VTA, GABAergic neurons account for 30.0% (Dobi et al., 2010) of total VTA neuron population. Furthermore, about 0.3 ± 0.1% of VTA GABAergic neurons co-express tyrosine hydroxylase, the marker of dopaminergic neurons, and 12.0 ± 0.6% of VTA GABAergic neurons were nitric oxide synthase-1 (NOS1)-positive (Yu et al., 2019). It is reported that VTA GABAergic neurons could inhibit nearby dopaminergic and/or glutamatergic neurons to affect reward processing and sleep (Creed et al., 2014; Yu et al., 2019). During general anesthesia, VTA GABAergic neurons are especially sensitive to anesthetics, manifesting as a reduced firing rate under adequate anesthesia, with their activity converted into phasic 0.5- to 2.0-s on/off periods (Lee et al., 2001). Along with the electrophysiological results, we proposed that isoflurane may act on VTA GABAergic neurons directly. These results are in accordance with sleep research in which VTA GABAergic neurons are non-rapid eye movement (NREM) sleep-promoting neurons, and ablation of these neurons elicits prolonged wakefulness, which persists for at least 4 months.

In contrast, VTA GABAergic neurons also have multiple long projections to a variety of brain areas associated with regulation of sleep and anesthesia (Creed et al., 2014), including the BF, preoptic area (POA), amygdala, mediodorsal thalamus, and LH, as well as arousal nuclei in the brainstem, such as the dorsal raphe and deep mesencephalic nuclei (Taylor et al., 2014). Yu et al. (2019) found that the LH, which is an important area that restricts the activity of the VLPO, receives extensive projections from VTA GABAergic neurons, and these projections play a role in NREM sleep induction (Yu et al., 2019). Our present study focused on the role of VTA GABA-LH projections in anesthesia. We found that optogenetic or chemogenetic activation of these projections exerted similar effects on anesthesia, including increased depth of anesthesia, facilitated induction, and delayed recovery, whereas inhibition of these terminals exerted contrasting effects on anesthesia. However, inhibition of VTA GABA-LH projections did not significantly reduce RORR. There may be two reasons for this observation. First, we considered that the VTA GABA-LH projections may mainly participate in anesthetic induction rather than emergence. The effect of VTA GABAergic neurons on emergence from anesthesia is mediated by their projections to other areas. Therefore, inhibition of the VTA–LH projections could temporarily alter EEG signals but may not be sufficient to accelerate behavioral recovery. Second, the VTA GABAergic neurons were already inhibited deeply by 1.4% isoflurane; therefore, further inhibition by optogenetic stimulation may not have been sufficient to induce a behavioral change.

Growing evidence suggests that anesthetic-induced neural oscillations underlie the primary mechanism of anesthetic action (Akeju and Brown, 2017). EEG spectral analysis showed that activation of both VTA GABAergic neurons and VTA GABA-LH projections increased delta wave but decreased gamma wave power percentages. Although the difference between two groups was not large, they were significant in statistics. Delta oscillations during anesthesia and sleep are partially mediated through cortical and thalamic hyperpolarization (Brown et al., 2012), which results from GABAergic inhibition of brainstem inputs to the thalamus and cortex (Schwartz and Kilduff, 2015). Gamma oscillations are usually implicated in conscious perception and are generated by cortical networks of fast-spiking interneurons targeting the cell bodies of glutamatergic neurons. Therefore, activation of VTA GABAergic neurons and VTA GABA-LH projections during anesthesia could aggravate hyperpolarization and induce neuronal network chaos, leading to amplification of delta oscillations and reduction of gamma oscillations, as observed in our own study during anesthesia. Other power bands were also affected by activation or inhibition of VTA GABAergic neurons or their LH protections; however, we considered these as a satellite phenomenon associated with frequency translation.

The current research did not define the target neuronal specificity in the LH innervated by VTA GABAergic neurons in general anesthesia regulation. The LH mainly contains a variety of neuronal subtypes expressing different chemical modulators, including orexin- and melanin-concentrating hormone (MCH)-producing neurons, of which orexin has a prominent role in regulating the stability of arousal. In the LH, about 60.0% of the VTA GABAergic targets are orexinergic neurons (Yu et al., 2019). Evidence shows that both isoflurane and sevoflurane inhibit wake-active orexinergic neurons (Kelz et al., 2008). Zhou et al. (2018) showed that chemogenetic activation of orexinergic neurons in the hypothalamus shortened emergence time from anesthesia (Zhou et al., 2018). These studies support a crucial role for LH orexinergic neurons in emergence from anesthesia; they also indicate that VTA GABA-LH projections regulating anesthesia and arousal may at least partially target orexinergic neurons. In sleep studies, evidence has shown that VTA GABAergic terminals could also inhibit wake-promoting GABAergic projection neurons in the LH (Herrera et al., 2016; Venner et al., 2016), which may explain their pro-anesthetic rather than an anti-emergence effect by inhibiting LH orexinergic neurons. The exact targets of VTA GABA-LH projections merit further exploration to better elucidate the details of the mechanisms of its action.

## Data Availability Statement

The datasets generated for this study are available on request to the corresponding author.

## Ethics Statement

The animal study was reviewed and approved by the Ethics Committee for Animal Experimentation of the Fourth Military Medical University.

## Author Contributions

LY conducted most of the research, analyzed the data, and drafted the manuscript. LL, YG, and SZ helped in conducting the research. HL, XZ, and DW helped to analyze the data. JD, HZ, and HD revised the manuscript. HZ helped to design the experiments. HD generated the idea, oversaw the whole experiments, and made the final approval of the manuscript.

## Conflict of Interest

The authors declare that the research was conducted in the absence of any commercial or financial relationships that could be construed as a potential conflict of interest.

## References

[B1] Akeju O. Brown E. N. (2017). Neural oscillations demonstrate that general anesthesia and sedative states are neurophysiologically distinct from sleep. *Curr. Opin. Neurobiol.* 44 178–185. 10.1016/j.conb.2017.04.011 28544930 PMC5520989

[B2] Brown E. N. Lydic R. Schiff N. D. (2010). General anesthesia, sleep, and coma. *N. Engl. J. Med.* 363 2638–2650. 10.1056/NEJMra0808281 21190458 PMC3162622

[B3] Brown R. E. Basheer R. McKenna J. T. Strecker R. E. McCarley R. W. (2012). Control of sleep and wakefulness. *Physiol. Rev.* 92 1087–1187. 10.1152/physrev.00032.2011 22811426 PMC3621793

[B4] Bruhn J. Bouillon T. W. Shafer S. L. (2000). Bispectral index (BIS) and burst suppression: revealing a part of the BIS algorithm. *J. Clin. Monit. Comput.* 16 593–596. 12580235 10.1023/A:1012216600170

[B5] Creed M. C. Ntamati N. R. Tan K. R. (2014). VTA GABA neurons modulate specific learning behaviors through the control of dopamine and cholinergic systems. *Front. Behav. Neurosci.* 8:8. 10.3389/fnbeh.2014.00008 24478655 PMC3897868

[B6] Cun Y. Tang L. Yan J. He C. Li Y. Hu Z. (2014). Orexin a attenuates the sleep-promoting effect of adenosine in the lateral hypothalamus of rats. *Neurosci. Bull.* 30 877–886. 10.1007/s12264-013-1442-8 24898402 PMC5562583

[B7] Dobi A. Margolis E. B. Wang H. L. Harvey B. K. Morales M. (2010). Glutamatergic and nonglutamatergic neurons of the ventral tegmental area establish local synaptic contacts with dopaminergic and nondopaminergic neurons. *J. Neurosci.* 30 218–229. 10.1523/JNEUROSCI.3884-09.2010 20053904 PMC3209506

[B8] Dong H. L. Fukuda S. Murata E. Zhu Z. Higuchi T. (2006). Orexins increase cortical acetylcholine release and electroencephalographic activation through orexin-1 receptor in the rat basal forebrain during isoflurane anesthesia. *Anesthesiology* 104 1023–1032. 10.1097/00000542-200605000-00019 16645455

[B9] Ferron J. F. Kroeger D. Chever O. Amzica F. (2009). Cortical inhibition during burst suppression induced with isoflurane anesthesia. *J. Neurosci.* 29 9850–9860. 10.1523/JNEUROSCI.5176-08.2009 19657037 PMC6666595

[B10] Franks N. P. (2008). General anaesthesia: from molecular targets to neuronal pathways of sleep and arousal. *Nat. Rev. Neurosci.* 9 370–386. 10.1038/nrn2372 18425091

[B11] Hapfelmeier G. Haseneder R. Eder M. Adelsberger H. Kochs E. Rammes G. (2001). Isoflurane slows inactivation kinetics of rat recombinant alpha1beta2gamma2L GABA(A) receptors: enhancement of GABAergic transmission despite an open-channel block. *Neurosci. Lett.* 307 97–100. 10.1016/s0304-3940(01)01950-4 11427309

[B12] Herrera C. G. Cadavieco M. C. Jego S. Ponomarenko A. Korotkova T. Adamantidis A. (2016). Hypothalamic feedforward inhibition of thalamocortical network controls arousal and consciousness. *Nat. Neurosci.* 19 290–298. 10.1038/nn.4209 26691833 PMC5818272

[B13] Hutt A. Buhry L. (2014). Study of GABAergic extra-synaptic tonic inhibition in single neurons and neural populations by traversing neural scales: application to propofol-induced anaesthesia. *J. Comput. Neurosci.* 37 417–437. 10.1007/s10827-014-0512-x 24976146 PMC4224752

[B14] Jiang-Xie L. F. Yin L. Zhao S. Prevosto V. Han B. X. Dzirasa K. (2019). A common neuroendocrine substrate for diverse general anesthetics and sleep. *Neuron* 102:1053-1065.e4. 10.1016/j.neuron.2019.03.033 31006556 PMC6554048

[B15] Kelz M. B. Sun Y. Chen J. Cheng M. Q. Moore J. T. Veasey S. C. (2008). An essential role for orexins in emergence from general anesthesia. *Proc. Natl. Acad. Sci. U.S.A.* 105 1309–1314. 10.1073/pnas.0707146105 18195361 PMC2234134

[B16] Kuki T. Fujihara K. Miwa H. Tamamaki N. Yanagawa Y. Mushiake H. (2015). Contribution of parvalbumin and somatostatin-expressing GABAergic neurons to slow oscillations and the balance in beta-gamma oscillations across cortical layers. *Front. Neural. Circ.* 9:6. 10.3389/fncir.2015.00006 25691859 PMC4315041

[B17] Lee R. S. Steffensen S. C. Henriksen S. J. (2001). Discharge profiles of ventral tegmental area GABA neurons during movement, anesthesia, and the sleep-wake cycle. *J. Neurosci.* 21 1757–1766. 10.1523/jneurosci.21-05-01757.2001 11222665 PMC6762953

[B18] Li J. Li H. Wang D. Guo Y. Zhang X. Ran M. (2019). Orexin activated emergence from isoflurane anaesthesia involves excitation of ventral tegmental area dopaminergic neurones in rats. *Br. J. Anaesth* 123 497–505. 10.1016/j.bja.2019.07.005 31399212

[B19] Moore J. T. Chen J. Han B. Meng Q. C. Veasey S. C. Beck S. G. (2012). Direct activation of sleep-promoting VLPO neurons by volatile anesthetics contributes to anesthetic hypnosis. *Curr. Biol.* 22 2008–2016. 10.1016/j.cub.2012.08.042 23103189 PMC3628836

[B20] Radtke F. M. Franck M. Lendner J. Kruger S. Wernecke K. D. Spies C. D. (2013). Monitoring depth of anaesthesia in a randomized trial decreases the rate of postoperative delirium but not postoperative cognitive dysfunction. *Br. J. Anaesth* 110(Suppl. 1), i98–i105. 10.1093/bja/aet055 23539235

[B21] Schwartz M. D. Kilduff T. S. (2015). The neurobiology of sleep and wakefulness. *Psychiatr. Clin. North Am.* 38 615–644. 10.1016/j.psc.2015.07.002 26600100 PMC4660253

[B22] Tan K. R. Yvon C. Turiault M. Mirzabekov J. J. Doehner J. Labouebe G. (2012). GABA neurons of the VTA drive conditioned place aversion. *Neuron* 73 1173–1183. 10.1016/j.neuron.2012.02.015 22445344 PMC6690362

[B23] Taylor N. E. Van Dort C. J. Kenny J. D. Pei J. Guidera J. A. Vlasov K. Y. (2016). Optogenetic activation of dopamine neurons in the ventral tegmental area induces reanimation from general anesthesia. *Proc. Natl. Acad. Sci. U.S.A.* 113 12826–12831. 10.1073/pnas.1614340113 27791160 PMC5111696

[B24] Taylor S. R. Badurek S. Dileone R. J. Nashmi R. Minichiello L. Picciotto M. R. (2014). GABAergic and glutamatergic efferents of the mouse ventral tegmental area. *J. Comp. Neurol.* 522 3308–3334. 10.1002/cne.23603 24715505 PMC4107038

[B25] Vanini G. Watson C. J. Lydic R. Baghdoyan H. A. (2008). Gamma-aminobutyric acid-mediated neurotransmission in the pontine reticular formation modulates hypnosis, immobility, and breathing during isoflurane anesthesia. *Anesthesiology* 109 978–988. 10.1097/ALN.0b013e31818e3b1b 19034094 PMC2743234

[B26] Venner A. Anaclet C. Broadhurst R. Y. Saper C. B. Fuller P. M. (2016). A novel population of wake-promoting GABAergic neurons in the ventral lateral hypothalamus. *Curr. Biol.* 26 2137–2143. 10.1016/j.cub.2016.05.078 27426511 PMC5160020

[B27] Westphalen R. I. Kwak N. B. Daniels K. Hemmings H. J. (2011). Regional differences in the effects of isoflurane on neurotransmitter release. *Neuropharmacology* 61 699–706. 10.1016/j.neuropharm.2011.05.013 21651920 PMC3130078

[B28] Yu X. Li W. Ma Y. Tossell K. Harris J. J. Harding E. C. (2019). GABA and glutamate neurons in the VTA regulate sleep and wakefulness. *Nat. Neurosci.* 22 106–119. 10.1038/s41593-018-0288-9 30559475 PMC6390936

[B29] Zecharia A. Y. Yu X. Gotz T. Ye Z. Carr D. R. Wulff P. (2012). GABAergic inhibition of histaminergic neurons regulates active waking but not the sleep-wake switch or propofol-induced loss of consciousness. *J. Neurosci.* 32 13062–13075. 10.1523/JNEUROSCI.2931-12.2012 22993424 PMC3466043

[B30] Zhang L. N. Li Z. J. Tong L. Guo C. Niu J. Y. Hou W. G. (2012). Orexin-A facilitates emergence from propofol anesthesia in the rat. *Anesth. Analg.* 115 789–796. 10.1213/ANE.0b013e3182645ea3 22798527

[B31] Zhang L. N. Yang C. Ouyang P. R. Zhang Z. C. Ran M. Z. Tong L. (2016). Orexin-A facilitates emergence of the rat from isoflurane anesthesia via mediation of the basal forebrain. *Neuropeptides* 58 7–14. 10.1016/j.npep.2016.02.003 26919917

[B32] Zhou W. Cheung K. Kyu S. Wang L. Guan Z. Kurien P. A. (2018). Activation of orexin system facilitates anesthesia emergence and pain control. *Proc. Natl. Acad. Sci. U.S.A.* 115 E10740–E10747. 10.1073/pnas.1808622115 30348769 PMC6233126

[B33] Zurek A. A. Bridgwater E. M. Orser B. A. (2012). Inhibition of alpha5 gamma-aminobutyric acid type a receptors restores recognition memory after general anesthesia. *Anesth. Analg.* 114 845–855. 10.1213/ANE.0b013e31824720da 22383672

